# Society Meetings

**Published:** 1906-06

**Authors:** 


### SOCIETY MEETINGS.

The Buffalo Academy of Medicine held meetings during April and May, as follows:

*Section of Pathology.*—Tuesday, April 17, 1906, at 8:30 P. M. (omitted from the list in May issue). Program: 1. Specimen of multiple diverticula of the jejunum, Irving P. Lyon. 2. Specimens from a case 2½ years after Talma's operation for cirrhosis of the liver; specimens also show cirrhotic carcinoma, Marshall Clinton and Charles A. Bentz. 3. Specimens from two cases of aplasia, James A. Gibson and David E. Wheeler. 4. Specimens of prehistoric wounds of bone, A. L. Benedict. 5. Remarks on recent investigations of uncinaria in Porto Rico, S. A. Dunham. 6. A fatal case of infection by bacillus ærogenes capsulatus; demonstration of the bacilli, Charles A. Bentz and H. K. DeGroat. 7. Exhibition of specimen of spirochetæ from a case with secondary lesions. 8. Organisms from a case of Vincent's angina.

*Section of Surgery.*—Tuesday, May 1, 1906, at 8:30 P. M. Program: The importance of the early recognition and operative treatment of malignant tumors, the variation of the extent of the operative removal according to the relative malignancy of the tumor, with lantern slide demonstration, Joseph C. Bloodgood, Baltimore, Md. Discussion by Roswell Park and H. R. Gaylord. Perforating typhoid ulcer, John Parmenter.

*Section of Medicine.*—Tuesday, May 8, 1906, at 8:30 P. M. Program: (a) The administration of iron, Eli H. Long. (b) Dropsy, Albert T. Lytle. Election of officers of the section.

*POSTPONEMENT OF MEETING.*—The regular meeting of the Pathological Section of the Academy, announced in the program for May 15, 1906, was postponed until a later date.

*Section of Obstetrics and Gynecology.*—Tuesday, May 22, 1906, at 8:30 P. M. Program: (a) Obstetrical amenities, Stephen Y. Howell. (b) Diagnosis of gonorrhea in the female, J. Henry Dowd. Election of officers of the section.

THE Medical Society of the County of Erie will hold its Eighty-fifth Semiannual Meeting at the Buffalo Society of Natural Sciences June 12, 1906 at 10 A. M. The object of the meeting in particular is to act upon the proposed by-laws that have been printed and sent to each member. The usual scientific program will be omitted on this account. Dr. A. H. Briggs is the president, and Dr. F. C. Gram the secretary. A full attendance is requested and urged.

---

THE Medical Society of the County of Steuben held its 89th Annual Meeting at Bath, Tuesday, May 8, 1906. The president, Dr. Frank H. Starr, of Corning, delivered an address, subject—"Entering into the Labor of Others;" after which papers were read as follows: Cancer, by William B. Jones, Rochester; Report of a case of Chronic Articular Rheumatism treated with Formic Acid, by Clayton K. Haskell, of the Soldier's Home Hospital, Bath; The Finsen *x*-ray in the Treatment of Diseases, by Frank W. Ross, Elmira; Steuben's Hills and Climate for the Treatment of Diseases, John A. Conway, Rexville; Memorial on the life of R. F. Parkhill, by L. M. Kysor, of Hornell; Memorial on the life of S. M. Switzer, by H. S. Gillette, Savona; Memorial on the life of Ira P. Smith, by H. R. Ainsworth, Addison. Officers for the current year as follows: president Charles O. Green, Hornell; vice-president, H. B. Smith, Corning; secretary and treasurer, W. W. Smith, Avoca.

